# Impact of rurality on diabetes screening in the US

**DOI:** 10.1186/s12889-019-7491-9

**Published:** 2019-09-26

**Authors:** Phoebe Tran, Lam Tran, Liem Tran

**Affiliations:** 1 Department of Chronic Disease Epidemiology, Yale University, 60 College Street, New Haven, CT 06510 USA; 2 Department of Biostatistics, Michigan School of Public Health, Ann Arbor, MI USA; 3 Deparment of Geography, University of Tennessee, Knoxville, TN USA

**Keywords:** Diabetes screening, Rurality, BRFSS; logistic regression; average adjusted predictions, Average marginal effects

## Abstract

**Background:**

Due to the high prevalence of diabetes risk factors in rural areas, it is important to identify whether differences in diabetes screening rates between rural and urban areas exist. Thus, the purpose of this study is to examine if living in a rural area, rurality, has any influence on diabetes screening across the US.

**Methods:**

Participants from the 2011, 2013, 2015, and 2017 nationally representative Behavioral Risk Factor Surveillance System (BRFSS) surveys who responded to a question on diabetes screening were included in the study (*n* = 1,889,712). Two types of marginal probabilities, average adjusted predictions (AAPs) and average marginal effects (AMEs), were estimated at the national level using this data. AAPs and AMEs allow for the assessment of the independent role of rurality on diabetes screening while controlling for important covariates.

**Results:**

People who lived in urban, suburban, and rural areas all had comparable odds (Urban compared to Rural Odds Ratio (OR): 1.01, Suburbans compared to Rural OR: 0.95, 0.94) and probabilities of diabetes screening (Urban AAP: 70.47%, Suburban AAPs: 69.31 and 69.05%, Rural AAP: 70.27%). Statistically significant differences in probability of diabetes screening were observed between residents in suburban areas and rural residents (AMEs: − 0.96% and − 1.22%) but not between urban and rural residents (AME: 0.20%).

**Conclusions:**

While similar levels of diabetes screening were found in urban, suburban, and rural areas, there is arguably a need for increased diabetes screening in rural areas where the prevalence of diabetes risk factors is higher than in urban areas.

## Background

As of 2015, approximately 23.1 million US adults have type 2 diabetes and 7.2 million have the disease but are undiagnosed [1]. The estimated yearly cost of diabetes on the US economy in terms of healthcare expenses and days of missed productivity is around $327 billion [2]. Type 2 diabetes is a serious health condition that can lead to complications such as cardiovascular disease, diabetic retinopathy, neuropathy, and chronic kidney disease (CKD) in addition to increasing the risks of mortality [3]. The distribution of type 2 diabetes is not uniform across the US as there is a 17% higher prevalence of diabetes in rural areas compared to urban areas [4].

In order to identify people with newly developed type 2 diabetes and offer them early treatment, the US Preventive Services Task Force recommends diabetes screening for people who are overweight or obese between the ages of 40–70 years [5]. The high prevalence of type 2 diabetes risk factors such as being overweight/obese (rural: 39.6%, urban: 33.4%), having high blood pressure (rural: 38.1%, urban: 32.6%), having high cholesterol (rural: 42.4%, urban: 38.8%), and being physically inactive (rural: 42.4%, urban: 38.8%) in rural areas is expected to lead to an increase in type 2 diabetes incidence in the coming years, making it important to assess diabetes screening levels in these places [6–8]. Although some work has been conducted on the levels of diabetes screening in the US, few studies have examined the impact of living in a rural area, rurality, on diabetes screening [9–13]. Existing work on levels of diabetes screening in rural areas has been limited by low sample sizes, consideration of only a small geographic area, and an inability to compare screening levels between rural and urban areas [12, 13].

In this study, the impact of rurality on US diabetes screening was examined using multiple years of data from a nationally representative dataset [14]. The intention of the study was to use marginal effects to isolate the independent role of rurality on diabetes screening while controlling for sociodemographic, clinical, and health seeking behavioral factors. This study’s results allowed for the identification of whether differences in diabetes screening exist between rural and urban areas.

## Methods

### Study sample

We used the 2011, 2013, 2015, and 2017 Centers for Disease Control and Prevention (CDC)‘s Behavioral Risk Factor Surveillance System (BRFSS) surveys as data [15–18]. The survey is an existing nationally representative questionnaire previously published elsewhere that collects information on health behaviors of the 50 states and the District of Columbia via both landline and cellphone from Genesys, Inc. phone lists [17, 19–22]. As BRFSS surveys are secondary publically available data, consent was not required for this study [21]. Not all residents have equal access to cellular devices so oversampling and raking adjustments are performed in the BRFSS to ensure representation of groups such as minorities and rural residents [14, 23]. We did not include data from BRFSS surveys prior to 2010 due to their usage of post-stratification weighting, which is incompatible with the iterative proportional fitting of more recent surveys. Furthermore, we did not include the 2012, 2014, and 2016 surveys because they did not necessarily include all variables we planned to adjust for in our analyses [15–18].

The data from the four surveys we did use were combined to maximize sample size and increase our study’s power. The study was comprised of survey participants >18y who responded to, “Had a test for high blood sugar or diabetes in the past three years?” (*n* = 1,889,712) [15–18]. This question was included in section 1.1 of the 2011, 2013, 2015, and 2017 BRFSS surveys [15–18]. Respondents who answered “don’t know/not sure” or refused to answer were excluded from the study. The exclusion of “don’t know/not sure” responses has precedent in CDC analyses of BRFSS data from Alaska, Montana, and North Carolina [24–26].

### Covariates

We wanted to adjust for covariates that have been associated with either differential rates of diabetes or diabetes screening. Sociodemographic factors influence access to preventative health care, so we included age, race, sex, education, income, and marital status as covariates [27–30]. Additionally, the American Diabetes Association (ADA) recommends that physicians screen adults that have a BMI ≥25 kg/m^2^ and have one or more risk factors such as hypertension (≥140/90 mmHg) and high cholesterol (HDL cholesterol level < 35 mg/dL, triglyceride level > 250 mg/dL), so we also included clinical (BMI, high blood pressure, high cholesterol, general health) and health behavior (health care coverage, personal doctor/health care provider) factors as covariates [30–32].

All covariates included in the study were categorized using the 2011, 2013, 2015, and 2017 BRFSS codebook groupings for that respective variable [15–18]. Here, we list the levels of the covariates that we adjusted for, all of which are categorical. For the sociodemographic factors: age (18 to 24, 25 to 34, 35 to 44, 45 to 54, 55 to 64, 65 or older), sex (Male, Female), household income (Less than $15,000, $15,000 to <$25,000, $25,000 to <$35,000, $35,000 to <$50,000, $50,000 or more), educational attainment (Never attended school or only kindergarten, Elementary, Some high school, High school graduate, Some college or technical school, College graduate), self-reported race (White, Black, Hispanic, Others (e.g., Asian, American Indian or Alaskan Native, Native Hawaiian or other Pacific Islander, other race, multiracial)), marital status (Married, Divorced, Widowed, Separated, Never married, A member of an unmarried couple). For the clinical factors: general health status (Excellent, Very good, Good, Fair, Poor), BMI categories (Underweight (BMI < 18.50), Normal Weight (18.50 < BMI < 25.00), Overweight (25.00 < BMI < 30.00), Obese (30.00 < BMI)), high blood pressure (“Have you ever been told by a doctor, nurse, or other health professional that you have high blood pressure?”) (Yes, No), high cholesterol levels (“Have you ever been told by a doctor, nurse, or other health professional that you have high cholesterol?”) (Yes, No). Finally, for the health behavior factors: health care coverage (Yes, No), and personal doctor/health care provider (Yes, only one, More than one, No) [15–18].

There are different ways to define/classify rurality for research or policy purposes [33–37]. In this case, the Metropolitan Status Codes (MSCODE) variable was the only variable in BRFSS datasets that could be used to define rurality [15–18]. Thus, the classification of rurality in this study was based on the information on Metropolitan Statistical Area (MSA) which is included in the MSCODE variable (in this context, e.g., using data that has already been collected, this study can be considered a post-hoc analysis) [15–18]. We define the definitions of rural and nonrural in accordance with the MSA taxonomy used by the Census Bureau and other government agencies for data analyses [35, 36]. That is, rural residents were defined as those living outside an MSA (MSCODE 5) whereas non-rural residents were all other respondents associated with the other MSCODE categories (MSCODE 1–4). We further divided the categorizations of non-rural residents as follows: urban residents were those who live in the center city of an MSA (MSCODE 1) whereas suburban residents were those who live outside the center city of an MSA but inside the county containing the center city (MSCODE 2) or those who live inside a suburban county of an MSA (MSCODE 3). We exclude results for residents of an MSA with no center city (MSCODE 4), because this MSCODE was only available for the 2012 and 2013 BRFSS surveys and the number of MSCODE4 individuals is small (*n* = 2901) in comparison to other categories within the same survey [15–18].

### Unadjusted screening levels

We calculated unadjusted diabetes screening levels in order to compare them to adjusted measures of diabetes screening. Crosstabulations were performed to generate unadjusted diabetes screening levels in each MSCODE. Crosstabulations were carried out in SAS 9.4 [38].

### Statistical models

We developed a logistic regression model to determine the relationship between diabetes screening rates and rurality while controlling for the sociodemographic, clinical, and health behavior factors discussed above. Survey weights were included in the model to account for the complex survey design and uneven weighting of survey data. The logistic model was run in SAS 9.4 [38]. As BRFSS data is readily available in the SAS format, it was a matter of convenience to run the logistic model in SAS [15–18]. We used a SAS procedure (Proc surveylogistic) for logistic models that included survey weights [38].

### Marginal probabilities

After fitting the logistic regression model, Average Adjusted Predictions (AAPs) and Average Marginal Effects (AMEs) were calculated [39, 40]. Stata 15 was used to calculate both AAPs and AMEs as SAS does not have a direct function for marginal probabilities like Stata does [41]. To calculate AAPs and AMEs in Stata, data in SAS were outputted into Stata format and proper codes were used to make the logistic regression in Stata take into account survey weights [41]. Next, we double checked results of the two logistic regressions from SAS and Stata and ensured they were the same. We then used the margins command to generate AAPS and AMEs from the logistic regression results in Stata [41].

We chose to calculate AAPs, a type of marginal probability, in addition to odds ratios due to their presentation in terms of absolute numerical values, which give the actual estimated testing probabilities and a more practical interpretation of testing differences [39, 40]. AAPs attempt to control for confounders by considering a hypothetical population with no variation in these factors; for example, an urban AAP is the predicted marginal probability of having been tested for high blood sugar/diabetes in the past 3 years if all respondents hypothetically resided in urban areas. For convenience, we also include the differences in marginal probabilities for diabetes testing in the past 3 years between a hypothetically all suburban or urban survey population and a hypothetically all rural one, known as average marginal effects or AMEs [39, 40].

## Results

People who responded to having had a test for high blood sugar or diabetes in the past 3 years were predominantly > 45 years (72.5%), female (58.4%), had an income >$25,000 (59.8%), were White (76.2%), had at least a high-school education (91.4%), reported that they felt good to excellent about their general health (80.5%), had healthcare coverage (90.0%), and a personal doctor (84.3%) (Table 1). BMI was roughly evenly split between normal weight (30.6%), overweight (33.7%), and obese (27.3%). More than half of respondents did not have high cholesterol (50.7%) but did have high blood pressure (59.5%). Most people reported either living in the center city of an MSA (22.1%) or not in an MSA at all (21.7%). Unadjusted diabetes screening prevalences were 67.2% for those in the center city of an MSA (MSCODE 1), 66.6% for those outside the center city of an MSA but inside the county containing the center city (MSCODE 2), 66.6% for those inside a suburban county of the MSA (MSCODE 3), 64.4% for those in an MSA that has no center city (MSCODE 4), and 64.7% for those not in an MSA (MSCODE 5).

**Table 1:** Sociodemographic, clinical, and health seeking behavioral factors among study participants in the 2011, 2013, 2015, and 2017 Behavioral Risk Factor Surveillance System surveys (*n* = 1,889,712)

Covariates	Had a test for high blood sugar or diabetes in the past 3 years	
	*n*	%
Age groups		
Age 18 to 24	100,689	5.3
Age 25 to 34	189,726	10.0
Age 35 to 44	228,777	12.1
Age 45 to 54	317,724	16.8
Age 55 to 64	420,192	22.2
Age 65 or older	632,604	33.5
Sex		
Male	785,750	41.6
Female	1,103,678	58.4
Refused	284	0.02
Household income		
Less than $15,000	183,236	9.7
$15,000 to < $25,000	277,192	14.7
$25,000 to < $35,000	179,594	9.5
$35,000 to < $50,000	231,180	12.2
$50,000 or more	718,954	38.1
Don’t know/Not Sure/Missing	299,556	15.9
Education		
Never attended school or only kindergarten	2567	0.1
Elementary	50,210	2.7
Some high school	102,724	5.4
High school graduate	538,144	28.5
Some college or technical school	515,485	27.3
College graduate	672,862	35.6
Don’t know/Not Sure/Missing	7720	0.4
Race		
White	1,440,751	76.2
Black	149,875	7.9
Hispanic	148,645	7.9
Others (e.g., Asian, American Indian or Alaskan Native, Native Hawaiian or other Pacific Islander, other race, multiracial)	119,579	6.3
Don’t know/Not Sure/Missing	30,862	1.6
Marital status		
Married	987,477	52.3
Divorced	262,439	13.9
Widowed	246,775	13.1
Separated	40,139	2.1
Never married	287,411	15.2
A member of an unmarried couple	53,181	2.8
Don’t know/Not Sure/Missing	12,290	0.7
General health		
Excellent	324,669	17.2
Very good	610,325	32.3
Good	586,084	31.0
Fair	257,366	13.6
Poor	104,880	5.6
Don’t know/Not Sure/Missing	2836	0.2
Body mass index (BMI) categories		
Underweight (BMI < 18.50)	30,199	1.6
Normal Weight (18.50 < BMI < 25.00)	578,862	30.6
Overweight (25.00 < BMI < 30.00)	637,026	33.7
Obese (30.00 < BMI)	516,432	27.3
Don’t know/Not Sure/Missing	127,193	6.7
High blood pressure		
Yes	1,124,597	59.5
No	759,569	40.2
Don’t know/Not Sure/Missing	5546	0.3
High cholesterol		
Yes	690,184	36.5
No	958,756	50.7
Don’t know/Not Sure/Missing	240,772	12.8
Health care coverage		
Yes	1,701,229	90.0
No	181,308	9.6
Don’t know/Not Sure/Missing	7175	0.4
Have personal doctor or health care provider		
Yes, only one	1,441,957	76.3
More than one	151,065	8.0
No	289,610	15.3
Don’t know/Not Sure/Missing	7080	0.4
Metropolitan Status Code		
In the center city of an MSA (MSCODE 1) (Unadjusted prevalence of high blood sugar or diabetes testing: 67.2%)	417,136	22.1
Outside the center city of an MSA but inside the county containing the center city (MSCODE 2) (Unadjusted prevalence of high blood sugar or diabetes testing: 66.6%)	231,483	12.3
Inside a suburban county of the MSA (MSCODE 3) (Unadjusted prevalence of high blood sugar or diabetes testing: 66.6%)	163,367	8.7
In an MSA that has no center city (MSCODE 4) (Unadjusted prevalence of high blood sugar or diabetes testing: 64.4%)	3901	0.2
Not in an MSA (MSCODE 5) (Unadjusted prevalence of high blood sugar or diabetes testing: 64.7%)	410,620	21.7
Don’t know/Not Sure/Missing	663,205	35.1

*MSA* Stands for metropolitan statistical area

Age, sex, household income, educational attainment, self-reported race, marital status, general health status, BMI, blood pressure, cholesterol levels, health care coverage, and personal doctor/health care provider were all associated with having had a test for high blood sugar or diabetes in the past 3 years in national level results (Table 2). Associations for all these covariates excluding sex were found to be statistically significant (*p* value< 0.05). Increasing income and education corresponded to increased odds of having had a test for high blood sugar or diabetes in the past 3 years. Black and Hispanic ethnicity were associated with higher odds of diabetes screening, but not White ethnicity. The odds of diabetes screening were similar among urban, suburban, and rural residents (Urban vs. Rural OR: 1.01, 95% CI: 0.98–1.04; Suburbans vs. Rural OR: 0.95, 0.94, 95% CI: 0.92–0.99, 0.90–0.98). Adjusted national level probabilities of diabetes screening were also comparable for urban, suburban, and rural individuals (urban (MSCODE 1) AAP: 70.47%; suburban areas (MSCODE 2 & MSCODE 3) AAPs: 69.31 and 69.05%, respectively; rural (MSCODE 5) AAP: 70.27%) (Table 3). Statistical differences in the adjusted probability of diabetes screening were only observed between people living in suburban areas (MSCODE 2 & MSCODE 3) and people in rural areas (MSCODE 5) (AMEs: − 0.96% and − 1.22%, respectively).

**Table 2:** Results of logistic regression models on diabetes screening

Parameters	Had a test for high blood sugar or diabetes in the past 3 years			
	Odds Ratio	95% Conf. Interval		*p*-value
		Lower	Upper	
Age (ref: Age 18 to 24)				
Age 25 to 34	1.57	1.37	1.80	< 0.0001
Age 35 to 44	1.44	1.27	1.64	< 0.0001
Age 45 to 54	1.81	1.59	2.06	< 0.0001
Age 55 to 64	2.34	2.05	2.66	< 0.0001
Age 65 or older	2.40	2.11	2.73	< 0.0001
Sex (ref: Female)				
Male	1.02	0.99	1.05	0.20
Household income (ref: Less than $15,000)				
$15,000 to <$25,000	1.09	1.02	1.17	0.01
$25,000 to <$35,000	1.13	1.05	1.21	0.001
$35,000 to <$50,000	1.17	1.10	1.26	< 0.0001
$50,000 or more	1.22	1.14	1.30	< 0.0001
Education (ref: High school graduate)				
Never attended school or only kindergarten	0.45	0.24	0.83	0.012
Elementary	0.77	0.70	0.85	< 0.0001
Some high school	0.81	0.75	0.87	< 0.0001
Some college or technical school	1.19	1.15	1.23	< 0.0001
College graduate	1.23	1.19	1.27	< 0.0001
Race (ref: White)				
Black	1.34	1.28	1.42	< 0.0001
Hispanic	1.28	1.16	1.40	< 0.0001
Others (e.g., Asian, American Indian or Alaskan Native, Native Hawaiian or other Pacific Islander, other race, multiracial)	1.00	0.94	1.07	0.94
Don’t know/Not sure/Refused	1.34	1.28	1.42	< 0.0001
General health (ref: Excellent)				
Very good	1.12	1.08	1.17	< 0.0001
Good	1.14	1.09	1.18	< 0.0001
Fair	1.23	1.17	1.30	< 0.0001
Poor	1.29	1.19	1.40	< 0.0001
BMI (ref: Normal Weight (18.50 < BMI < 25.00))				
Underweight (BMI < 18.50)	0.89	0.81	0.98	0.016
Overweight (25.00 < =BMI < 30.00)	1.29	1.25	1.33	< 0.0001
Obese (BMI > =30.00)	1.78	1.72	1.85	< 0.0001
High blood pressure (ref: No)				
Yes	0.71	0.69	0.73	< 0.0001
High cholesterol (ref: No)				
Yes	1.10	1.07	1.13	< 0.0001
Health care coverage (ref: No)				
Yes	1.51	1.42	1.60	< 0.0001
Personal doctor/health care provider (ref: No)				
Yes, only one	2.04	1.95	2.13	< 0.0001
More than one	1.86	1.75	1.97	< 0.0001
Marital status (ref: Married)				
Divorced	1.00	0.96	1.05	0.879
Widowed	0.90	0.87	0.94	< 0.0001
Separated	1.08	0.97	1.19	0.153
Never married	0.91	0.87	0.97	0.001
A member of an unmarried couple	1.15	1.04	1.27	0.005
Metropolitan Status Code (ref: Not in an MSA)				
In the center city of an MSA	1.01	0.98	1.04	0.534
Outside the center city of an MSA but inside the county containing the center city	0.95	0.92	0.99	0.011
Inside a suburban county of the MSA	0.94	0.90	0.98	0.002

Model adjusted for Age, Sex, Household income, Education, Race, General health, BMI, High blood pressure, High cholesterol, Health care coverage, Personal doctor/health care provider, Marital status, and Metropolitan Status Code

Significant at 0.05 level (2-tailed)

MSCODE 5

MSCODE 1

MSCODE 2

MSCODE 3

**Table 3:** Average adjusted predictions (AAPs) for different Metropolitan Status Codes (MSCODEs) and Average marginal effects (AMEs) of MSCODE 1, 2, 3 vs. 5

Parameters	Had a test for high blood sugar or diabetes in the past 3 years			
	Estimate (%)	95% Conf. Interval		*p*-value
		Lower	Upper	
Metropolitan Status Code	Average Adjusted Predictions (AAPs)			
In the center city of an MSA (MSCODE 1) – urban	70.47	70.04	70.91	< 0.0001
Outside the center city of an MSA but inside the county containing the center city (MSCODE 2) – suburban	69.31	68.73	69.89	< 0.0001
Inside a suburban county of the MSA (MSCODE 3) – suburban	69.05	68.45	69.66	< 0.0001
Not in an MSA (MSCODE 5) – rural	70.27	69.81	70.73	< 0.0001
Metropolitan Status Code	Average Marginal Effects (AMEs) (ref: Not in an MSA (MSCODE 5) – rural)			
In the center city of an MSA (MSCODE 1) – urban	0.20	−0.44	0.84	0.534
Outside the center city of an MSA but inside the county containing the center city (MSCODE 2) – suburban	−0.96	−1.71	−0.22	0.011
Inside a suburban county of the MSA (MSCODE 3) – suburban	−1.22	−1.98	−0.46	0.002

AAPs are a type of marginal probability that attempt to control for the other sociodemographic, clinical, and health seeking behavioral factors by considering a hypothetical respondent population with no variation in these factors calculated [39, 40]

AMEs are the differences in AAPs

## Discussion

We conducted a nationally representative study using the 2011, 2013, 2015, and 2017 BRFSS to examine the influence of rurality on diabetes screening. Across the US, urban, suburban, and rural residents had similar odds and adjusted probabilities of diabetes screening. Urban residents (MSCODE 1) had similar odds and probability of diabetes screening compared to rural residents (MSCODE 5). There was a larger difference in probability of diabetes screening between suburban residents (MSCODE 2 and MSCODE 3) and rural residents than between urban and rural residents.

Previous work on the influence of rurality on diabetes screening has tended to focus on this research question in only a small number of study participants over a limited geographic area [12, 13]. A study of diabetes screening in 540 people in 12 rural West Virginian counties found that 61.8% of them were at high risk for diabetes, but did not examine what factors influence diabetes screening in this population [13]. In a study using 2009 and 2010 BRFSS data to examine diabetes screening at the county level in 11 largely rural Appalachian states, study participants > 65 years in low economic status counties had a 8.1% lower screening rate compared to those from high economic status counties [12]. However, neither of these two studies compared levels of diabetes screening between rural and urban areas which makes it hard to detect the existence of rural-urban disparities in diabetes screening [12, 13]. It is difficult to say whether the results of this study are consistent with the West Virginian and Appalachian studies as these two studies addressed the prevalence of diabetes in rural study participants who had never had a diabetes diagnosis rather than the direct influence of rurality on diabetes screening [12, 13].

The similar levels of diabetes screening seen between rural and urban areas may be the product of (1) overall higher levels of diabetes screening by all physicians and/or (2) the need for rural residents to travel to urban hospitals for diabetes prevention and treatment [12, 16, 18, 42]. With the implementation of more definitive diabetes screening guidelines by the US Preventive Services Task Force in 2014, the prevalence of diabetes screening has increased from 46.80% in 2010 to 54.66% in 2015 and 55.02% in 2017 [16, 43]. These figures for national uptake of diabetes screening (46.80% in 2010 to 54.66% in 2015 and 55.02% in 2017) are taken directly from the BRFSS and represent diabetes screening prevalence that has not been adjusted for any sociodemographic, clinical, and health seeking behavioral factors in these years while the predicted values in Table 3 have been adjusted for the sociodemographic, clinical, and health seeking behavioral factors mentioned in the Methods section of this study. Confounding from these factors may be one explanation as to why figures for national uptake of diabetes screening seem much lower than the predicted values in Table 3. In addition, people who responded “Don’t know/Not Sure” as well as “Refused” were included in the calculation of these national figures while they were not included when calculating the predicted values in Table 3. Another possible explanation for the differences between the figures for national uptake of diabetes screening and the predicted values in Table 3 was that records with missing data in any covariates were removed in the logistic regression models. A lack of medical facilities and physicians in rural communities often forces rural residents to travel to urban hospitals in order to receive medical care [18, 42, 44]. A study of rural residents in New York found that many, especially those in counties with few medical resources, had to travel large distances in order to receive care from a primary physician [44]. As a result, many rural residents end up visiting doctors at urban hospitals whose referral rates for diabetes screening are similar for both rural and urban residents, leading to similar levels of diabetes screening for these two groups [44, 45]. However, as a large number of rural residents do not have health insurance or are unable to travel to urban hospitals, the underlying number of rural residents with diabetes risk factors that are not receiving but need diabetes screening may be underestimated [7, 8, 42, 44].

The main limitation of this study revolves around its usage of self-reported BRFSS survey data, allowing for the possibility of survey responses not accurately true national rates. However, several past BRFSS validation studies have shown high correlation between BRFSS responses and in-person measurements of clinical factors, with R^2^ estimates between 89 and 92% [46, 47]. Another study using Massachusetts BRFSS response values found that their percentage difference with “true” Massachusetts EHR data was less than 5% for diabetes and obesity and 15% for hypertension [48]. It is likely that the effects of self-reported bias with respect to accuracy are minor to negligible, with at most minimal levels of non-differential misclassification bias. Our decision to focus solely on diabetes screening at the national level could obscure regional differences in diabetes screening. While a large array of sociodemographic, clinical, and health seeking behavioral factors was controlled for in the analyses, some residual confounding may still remain [49]. We are unable to control for health beliefs and literacy as well as the behavior of healthcare providers since the BRFSS does not contain this information. However, many covariates associated with diabetes and health screening behavior that are available in the BRFSS surveys were included in the analyses and adjusted for in the study [27–30].

We highlight some advantages of this study relative to past research examining the influence of rurality on diabetes screening. By combining four editions of the BRFSS surveys, this nationwide study has a large study population and enough statistical power to account for several sociodemographic, clinical, and health seeking behavioral factors that were not considered in aggregate in previous research. There is less variance in our estimates within the different MSCODEs due to the usage of survey weights and oversampling in the study population, and our reporting of marginal effects allows for direct comparisons of screening probabilities (especially for factors where a reference level for odds ratio is not obvious). Finally, an ordinal scale that separates areas by rurality is immediately apparent from the usage of MSCODE.

## Conclusions

This study sought to determine if there are disparities in diabetes screening between suburban/urban and rural residents across the US. While levels of diabetes screening were similar for rural, suburban, and urban residents, the slightly lower prevalence of diabetes screening in rural vs. urban residents suggests a need for increased diabetes screening in rural populations that have a higher prevalence of diabetes risk factors than urban populations, are underserved, and oftentimes lack knowledge and resources with respect to diabetes [7, 8, 42]. Additional diabetes screening efforts in rural areas will increase the number of diabetes cases that can be detected early, improve the lives of individuals who have been living with undiagnosed diabetes via prompt treatment, and ultimately reduce serious diabetes complications that can arise if the condition is left untreated [2, 3].

## Data Availability

The datasets generated and/or analyzed during the current study are publicly available at the CDC’s website, [https://www.cdc.gov/brfss/annual_data/annual_2011.htm, https://www.cdc.gov/brfss/annual_data/annual_2013.html, https://www.cdc.gov/brfss/annual_data/annual_2015.html, https://www.cdc.gov/brfss/annual_data/annual_2017.html].

## Abbreviations

AAP: Average adjusted predictions
AME: Average marginal effects
BMI: Body mass index
BRFSS: Behavioral Risk Factor Surveillance System
CDC: Centers for Disease Control and Prevention
CKD: Chronic kidney disease
MSA: Metropolitan Statistical Area
MSCODE: Metropolitan Status Codes
